# Prevalence of Metabolic Dysfunction-Associated Steatotic Liver Disease Among Overweight and Obese Patients With Type 2 Diabetes Mellitus

**DOI:** 10.7759/cureus.99670

**Published:** 2025-12-19

**Authors:** Priyanka Budhwani, Bikas Goswani, Somiddho Debnath, Aparna Shukla

**Affiliations:** 1 Internal Medicine, Howrah District Hospital, Howrah, IND; 2 General Medicine, Howrah District Hospital, Howrah, IND; 3 Biotechnology, King George's Medical University, Lucknow, IND

**Keywords:** eastern india, glycemic control, hepatic steatosis (masld), hypercholesterolemia, metabolic-associated fatty liver disease, metabolic dysfunction-associated steatotic liver disease, non-alcoholic fatty liver disease, obesity, prevalence, type 2 diabetes mellitus

## Abstract

Background

Metabolic dysfunction-associated steatotic liver disease (MASLD), earlier known as non-alcoholic fatty liver disease (NAFLD), is the most common chronic liver disease globally, strongly associated with type 2 diabetes mellitus (T2DM) and metabolic syndrome. Its prevalence in India is increasing due to urbanization, sedentary lifestyles, and dietary changes. NAFLD/MASLD is common among individuals with T2DM, yet it often remains underdiagnosed in resource-limited settings. This study aimed to estimate the prevalence of MASLD among T2DM patients in an urban district hospital in Howrah, India, and assess its association with demographic, anthropometric, glycemic, lipid, and liver enzyme profiles.

Methods

This cross-sectional observational study was conducted in the Department of General Medicine, District Hospital, Howrah, from November 2018 to October 2019. A total of 150 purposively sampled adults (20-75 years) with T2DM (per ADA 2017 criteria) and BMI ≥25 kg/m² were enrolled after excluding significant alcohol use, viral hepatitis, chronic liver disease, HIV, and hepatotoxic drug use. Data collected included demographics, BMI, lifestyle factors, fasting blood sugar (FBS), postprandial blood sugar (PPBS), HbA1c, lipid profile, and liver function tests. Abdominal ultrasonography, performed by a single blinded radiologist, was used to diagnose MASLD based on standard echogenic criteria. Statistical analysis included independent t-tests, Chi-square tests, and multivariate logistic regression to identify predictors of MASLD, with p<0.05 considered significant.

Results

Of 150 T2DM patients (mean age: 54.3 ± 10.2 years; 41.3% male), MASLD prevalence was 34% (n=51). MASLD patients had significantly higher BMI (29.10 ± 3.12 vs. 26.96 ± 2.87 kg/m²; p < 0.001) and obesity rates (33.3% vs. 7.1%). Poor glycemic control was more common in MASLD: elevated FBS (≥140 mg/dL) in 60.8% vs. 26.3%, PPBS (≥200 mg/dL) in 76.5% vs. 40.4%, and HbA1c ≥7.5% in 47.1% vs. 23.4% (all p < 0.01). Elevated ALT (≥45 IU/L) occurred in 17.6% vs. 6.1% (p = 0.035) and AST (≥45 IU/L) in 51% vs. 19.2% (p < 0.001). Hypercholesterolemia (≥200 mg/dL) was more frequent in MASLD (47.1% vs. 14.1%, p < 0.001), whereas low HDL was prevalent in both groups. Logistic regression identified BMI ≥30 kg/m² (aOR 4.29; 95% CI 1.88-9.78), poor glycemic control (HbA1c ≥7.5%; aOR 2.67; 95% CI 1.21-5.89), and hypercholesterolemia (aOR 3.41; 95% CI 1.54-7.54) as independent predictors.

Conclusions

MASLD affects over one-third of urban T2DM patients in this Eastern Indian cohort, with strong associations to obesity, poor glycemic control, elevated liver enzymes, and hypercholesterolemia. Given its asymptomatic course and potential progression to cirrhosis and cardiovascular disease, routine MASLD screening in diabetic care is warranted. Early lifestyle and metabolic interventions could reduce long-term morbidity.

## Introduction

Metabolic dysfunction-associated steatotic liver disease (MASLD), previously referred to as non-alcoholic fatty liver disease (NAFLD), is currently the most prevalent chronic liver disease worldwide, affecting approximately 10-30% globally, with rates rising alongside the global epidemic of obesity and metabolic syndrome [1]. In accordance with recent international consensus on fatty liver disease nomenclature, MASLD replaces the earlier term NAFLD for improved clarity. The condition encompasses a histological spectrum from benign hepatic steatosis to metabolic dysfunction-associated steatohepatitis (MASH), fibrosis, and eventually cirrhosis, which now constitutes a leading indication for liver transplantation in many Western countries [2]. MASLD is increasingly recognized not merely as a hepatic disorder but as a systemic disease, strongly associated with type 2 diabetes mellitus (T2DM), cardiovascular disease, and chronic kidney disease [3]. Insulin resistance, a hallmark of T2DM, promotes hepatic fat accumulation through increased flux of free fatty acids (FFA) to the liver. In turn, MASLD exacerbates systemic inflammation via pro-inflammatory cytokines such as tumor necrosis factor-alpha (TNF-α) and Interleukin 6 (IL-6), and adipokines like leptin and adiponectin [4]. Although up to 70% of individuals with T2DM may have MASLD [5], it remains underdiagnosed due to its asymptomatic nature and the lack of standardized screening protocols, especially in resource-constrained settings.

MASLD prevalence is high across industrialized nations (30-40% in the U.S. and Europe) and rising sharply in Asian countries like India, where urbanization and lifestyle changes have fueled metabolic disorders [6]. The pathogenesis of MASLD has evolved from the original “two-hit hypothesis” to models incorporating multiple parallel hits, including lipotoxicity, mitochondrial dysfunction, impaired hepatocyte regeneration, and gut-liver axis dysregulation [7-9]. Diagnostic limitations persist; while liver biopsy remains the gold standard, non-invasive imaging tools like ultrasound-despite operator dependency-offer practical screening alternatives in low-resource environments. Given this backdrop, the present study aims to estimate the prevalence of MASLD among patients with T2DM attending a district hospital in Howrah, India, using ultrasound imaging, and to explore its association with demographic and clinical parameters.

## Materials and methods

Ethical considerations

The study was conducted following the ethical principles outlined in the Declaration of Helsinki. Ethical approval was obtained from the Institutional Ethics Committee of District Hospital, Howrah (Approval ID: IEC/DH/Howrah/2018/11). All participants provided written informed consent prior to inclusion. Data confidentiality and anonymity were ensured throughout the study.

Study design and setting

**Figure 1:**
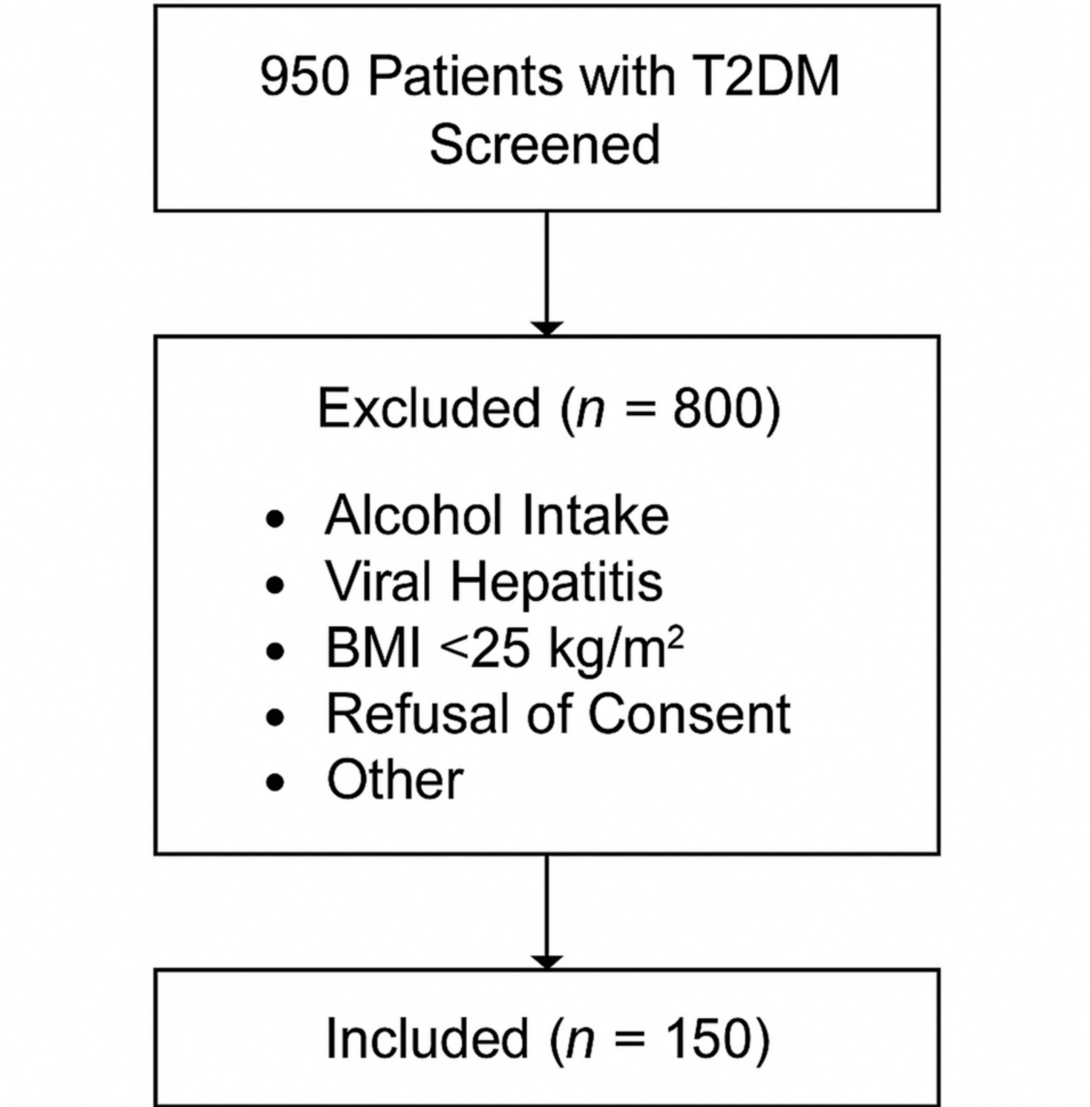
Flowchart of participant screening and enrollment

This was a cross-sectional observational study with prospective recruitment of participants, conducted in the Department of General Medicine, District Hospital, Howrah, West Bengal, India, over a 12-month period from November 2018 to October 2019. The aim was to determine the prevalence of MASLD among individuals with T2DM attending the outpatient department (OPD) of the hospital. A total of 950 patients were screened. Of these, 800 were excluded due to alcohol intake, viral hepatitis, BMI <25 kg/m², refusal of consent, or other exclusion criteria. Finally, 150 patients were included. Purposive sampling was used because only patients meeting strict metabolic criteria (BMI ≥25 kg/m² with confirmed T2DM) were eligible. This non-probability approach may introduce selection bias. A participant flowchart has been added to show the screening, exclusion, and final recruitment process. Recruitment was consecutive among eligible patients during the study period (Figure 1).

The study was based on data collected from November 2018 to October 2019 because this period represented the most recent complete dataset available at the time of study initiation, and allowed adequate follow-up for ethical clearance and data validation.

Inclusion and exclusion criteria

Inclusion Criteria

Participants were recruited if they were adults between 20 and 75 years of age with a confirmed diagnosis of T2DM based on the American Diabetes Association (ADA) 2017 guidelines [10]. This diagnosis could be established through fasting plasma glucose levels of 140 mg/dL or higher, a two-hour post-load plasma glucose of at least 200 mg/dL during an oral glucose tolerance test, or an HbA1c value of 6.5% or greater. The cutoffs of FBS ≥140 mg/dL and PPBS ≥200 mg/dL were based on established glycemic thresholds for uncontrolled T2DM (ADA 2017) [10]. In addition, eligible individuals were required to have a body mass index (BMI) of 25 kg/m² or more, in accordance with the World Health Organization (WHO) Asian criteria for overweight and obesity. BMI ≥25 kg/m² was intentionally used to focus on overweight and obese diabetics who are at higher biological risk for MAFLD. Only those who agreed to undergo abdominal ultrasonography and provided written informed consent were considered for participation.

Exclusion Criteria

Patients were excluded if they reported alcohol intake exceeding 30 grams per day in men or 20 grams per day in women, which was assessed using a structured alcohol use questionnaire including the CAGE screening tool. Individuals with serological evidence of viral hepatitis (HBsAg or anti-HCV positivity), HIV infection, or a history of recent or past use of hepatotoxic medications such as methotrexate or corticosteroids were also excluded. Furthermore, patients with a previous history of chronic liver disease, jaundice, or cirrhosis were not enrolled in the study.

Participant recruitment and data collection

Eligible patients were identified from the general medicine OPD through their diabetes clinic records. Those meeting the inclusion criteria were invited to participate after providing informed consent. All data were collected using a structured case record form.

Clinical and Anthropometric Assessment

Demographic data, including age, sex, and BMI, were recorded. A structured questionnaire captured lifestyle habits (including alcohol intake), medication use, and past medical history.

Laboratory Investigations

After a minimum 10-hour overnight fast, venous blood samples were collected and analyzed for:

Fasting plasma glucose (FPG): Hexokinase method.

Glycated hemoglobin (HbA1c): Ion-exchange high-performance liquid chromatography (HPLC).

Lipid profile: Enzymatic colorimetric assays were used to estimate total cholesterol, triglycerides (TG), high-density lipoprotein cholesterol (HDL-C), and low-density lipoprotein cholesterol (LDL-C).

Liver function tests (LFTs): Included aspartate aminotransferase (AST), alanine aminotransferase (ALT), alkaline phosphatase (ALP), and serum bilirubin (total and direct), measured by standardized enzymatic kinetic assays.

All assays were conducted in the hospital’s central laboratory with internal and external quality control measures in place. Manufacturer-recommended reference ranges were used for interpretation.

Ultrasonography for MASLD Diagnosis

All participants underwent abdominal ultrasonography using a Mindray DC-7 machine with a 5 MHz convex probe. The USG was performed after 8-10 hours of fasting by a single experienced radiologist blinded to clinical and laboratory parameters to reduce observer bias.

MASLD was diagnosed based on standard echogenic criteria requiring at least two of the following: Increased hepatorenal echogenicity contrast; Blurring of hepatic vascular margins; Posterior beam attenuation (deep attenuation); No liver biopsies were performed due to the non-invasive nature of the study.

Statistical analysis

All data were entered and analyzed using IBM SPSS Statistics Version 22.0. Continuous variables were tested for normality using the Kolmogorov-Smirnov test. Variables with normal distribution were expressed as mean ± standard deviation (SD), and comparisons between MASLD and non-MASLD groups were made using the independent samples t-test. Categorical variables were presented as frequencies and percentages, with comparisons made using the Chi-square test.

To identify independent predictors of MASLD among T2DM patients, multivariate logistic regression analysis was performed. Covariates included in the model were age, BMI, lipid parameters, and HbA1c levels-based on biological plausibility and univariate significance. Results were expressed as adjusted odds ratios (aOR) with 95% confidence intervals (CI). A p-value <0.05 was considered statistically significant. Missing data were handled using pairwise deletion where appropriate.

## Results

Demographic and anthropometric profile of study participants

A total of 150 patients with T2DM were enrolled in this study, including 62 males (41.3%) and 88 females (58.7%). The mean age of the participants was 54.3 ± 10.2 years. Among them, 34% (n = 51) were diagnosed with MASLD based on ultrasonographic findings, while the remaining 66% (n = 99) did not have MASLD.

**Table 1:** Demographic characteristics of participants with and without MASLD *Statistically significant (p < 0.05) MASLD: Metabolic dysfunction-associated steatotic liver disease

Parameter	Category	MASLD (n = 51)	Non-MASLD (n = 99)	p-value
Sex	Male	26 (51.0%)	36 (36.4%)	0.082
	Female	25 (49.0%)	63 (63.6%)	
Age (years)	20–29	2 (3.9%)	0 (0.0%)	0.021*
	30–39	1 (1.9%)	4 (4.0%)	
	40–49	16 (31.3%)	24 (24.2%)	
	50–59	13 (25.4%)	50 (50.5%)	
	60–69	15 (29.4%)	17 (17.2%)	
	≥70	4 (7.8%)	4 (4.0%)	
BMI (kg/m²)	25.0–29.9	33 (64.7%)	91 (91.9%)	<0.001*
	30.0–34.9	17 (33.3%)	7 (7.1%)	
	≥35.0	1 (2.0%)	1 (1.0%)	

MASLD was more frequently observed in males 26 (51%) than in females 25 (49%), although this sex difference did not reach statistical significance (p = 0.082). The highest prevalence of MASLD was seen in the 40-49 years 16 (31.3%) and 60-69 years 15 (29.4%) age groups. Notably, obesity (BMI ≥30 kg/m²) was significantly more common in the MASLD group (33.3%) compared to the non-MASLD group (7.1%) (p < 0.001), suggesting a strong association between elevated BMI and MASLD (Table 1).

Glycemic control among MASLD and non-MASLD groups

A significantly higher proportion of MASLD patients had poor glycemic control. In 31 (60.8%) of patients in the MASLD group had elevated FBS (≥140 mg/dL) compared to 26 (26.3%) in the non-MASLD group. Similarly, 39 (76.5%) of MASLD patients had PPBS ≥200 mg/dL, compared to 40 (40.4%) in the non-MASLD group. Also, 24 (47.1%) of MASLD patients had HbA1c ≥7.5%, compared to 23 (23.4%) in non-MASLD patients. All differences were statistically significant, indicating a strong association between MASLD and poor glycemic control (Table 2).

**Table 2:** Association between MASLD status and glycemic parameters *Statistically significant (p < 0.05) MASLD: Metabolic dysfunction-associated steatotic liver disease

Parameter	Range	MASLD (n = 51)	Non-MASLD (n = 99)	p-value
Fasting Blood Sugar (FBS)	<140 mg/dL	20 (39.2%)	73 (73.7%)	<0.001*
	≥140 mg/dL	31 (60.8%)	26 (26.3%)	
Postprandial Blood Sugar (PPBS)	<200 mg/dL	12 (23.5%)	59 (59.6%)	<0.001*
	≥200 mg/dL	39 (76.5%)	40 (40.4%)	
HbA1c	<7.5%	27 (52.9%)	76 (76.6%)	0.004*
	≥7.5%	24 (47.1%)	23 (23.4%)	

Liver enzyme profile among MASLD and non-MASLD groups

Elevated ALT (SGPT) and AST (SGOT) levels were significantly more common among MASLD patients. In 9 (17.6%) of patients with MASLD had ALT ≥45 IU/L, compared to 6 (6.1%) in the non-MASLD group (p = 0.035). More strikingly, 26 (51%) of MASLD patients had AST ≥45 IU/L, compared to only 19 (19.2%) of those without MASLD (p < 0.001), suggesting a significant liver enzyme elevation associated with MASLD (Table 3).

**Table 3:** Comparison of liver enzymes (ALT and AST) between MASLD and non-MASLD participants. *Statistically significant (p < 0.05) AST (SGOT): Aspartate aminotransferase (Serum Glutamic-Oxaloacetic Transaminase); ALT (SGPT): Alanine transaminase (Serum Glutamic-Pyruvic Transaminase); MASLD: Metabolic dysfunctional-associated steatotic liver disease.

Parameter	Range (IU/L)	MASLD (n = 51)	Non-MASLD (n = 99)	p-value
ALT (SGPT)	<45 IU/L	42 (82.4%)	93 (93.9%)	0.035*
	≥45 IU/L	9 (17.6%)	6 (6.1%)	
AST (SGOT)	<45 IU/L	25 (49.0%)	80 (80.8%)	<0.001*
	≥45 IU/L	26 (51.0%)	19 (19.2%)	

Lipid profile and MASLD association

In 47.1% of MASLD patients had total cholesterol ≥200 mg/dL, compared to only 14 (14.1%) in the non-MASLD group (p < 0.001), indicating a strong association between hypercholesterolemia and MASLD. Interestingly, low HDL cholesterol levels were common in both groups, but slightly more prevalent in the non-MASLD group 75 (75.8%), possibly reflecting widespread dyslipidemia in the diabetic population (Table 4).

**Table 4:** Comparison of lipid profile (Total Cholesterol and HDL) between MASLD and non-MASLD groups. *Statistically significant (p < 0.05) MASLD: Metabolic dysfunction-associated steatotic liver disease; HDL: High-Density Lipoprotein

Parameter	Range	MASLD (n = 51)	Non-MASLD (n = 99)	p-value
Total Cholesterol	<200 mg/dL	27 (52.9%)	85 (85.9%)	<0.001*
	≥200 mg/dL	24 (47.1%)	14 (14.1%)	
HDL Cholesterol	M <40 / F <50 mg/dL	26 (50.9%)	75 (75.8%)	0.003*
	M ≥40 / F ≥50 mg/dL	25 (49.1%)	24 (24.2%)	

Summary of key findings

The study found that 34% of patients with T2DM had MASLD. The condition was more frequent among men, individuals in middle-to-older age groups, and those with obesity. Patients with MASLD showed significantly poorer glycemic control, higher liver enzyme levels, and elevated total cholesterol compared to non-MASLD participants. Low HDL levels were prevalent across both groups, suggesting widespread dyslipidemia in this population.

## Discussion

In this hospital-based cross-sectional observational study involving 150 T2DM patients in an urban setting of West Bengal, we observed a 34% prevalence of MASLD diagnosed by ultrasonography. This finding reflects a significant burden of hepatic steatosis among diabetic individuals in this region and supports the hypothesis that urbanization, sedentary lifestyles, and nutritional transitions are key contributors to the rising MASLD prevalence in India. Our cohort’s mean age and BMI also correspond with a high-risk metabolic profile, further amplifying disease burden.

Prevalence of MASLD and regional comparisons

Among our MASLD cases, 51% were male and 49% female, indicating a near-equal gender distribution. While our prevalence of 34% aligns with other Indian studies, such as Mohan et al. (2009), who reported a 32% NAFLD prevalence in an urban South Indian diabetic population [11], and it exceeds the 24.5% rate found in Eastern India by Das et al. (2010) [12]. These discrepancies likely reflect differences in methodology (hospital-based vs. population-based studies), diagnostic tools (ultrasound vs. biopsy), demographic composition, and lifestyle patterns. For example, our urban cohort-primarily comprising Muslim individuals, exhibits higher meat consumption and central adiposity, two known contributors to insulin resistance and hepatic lipid accumulation. However, any such assumptions must be cautiously interpreted and should ideally be substantiated with explicit dietary intake data.

Nationally, NAFLD prevalence varies across regions. In North India, a recent study by Asadullah et al. (2022) reported a 65.7% prevalence [13], while previously Amarapurkar et al. (2007) estimated NAFLD in 30-90% of diabetic patients in Western India [14]. These wide variations underscore the role of ethnicity, dietary practices, socioeconomic status, and degree of urbanization in influencing NAFLD risk.

Glycemic control and MASLD

Our study found significantly poorer glycemic control in patients with MASLD. Mean fasting blood glucose (218.21 mg/dL), postprandial blood glucose (244.58 mg/dL), and HbA1c (7.93%) were all significantly elevated in the NAFLD group compared to their non-MASLD counterparts (FBS: 182.4 mg/dL; PPBS: 208.93 mg/dL; HbA1c: 7.29%; p < 0.01 for all). These findings reinforce the strong interconnection between chronic hyperglycemia and hepatic steatosis. The observed association aligns with recent findings by Ziolkowska et al. (2021), who showed that insulin resistance leads to excessive free fatty acid influx into the liver, promoting triglyceride accumulation and hepatocellular injury [15]. Another recent study by Alexopoulos et al. (2021) similarly concluded that poor glycemic control is a predictive factor for NAFLD severity [16]. However, our findings diverge from Xie et al. (2022), who were unable to find the association between HbA1c and hepatic steatosis [17]

Gender distribution patterns

Although previous literature reports gender-linked variations in MASLD, our study did not find a statistically significant difference (p = 0.082). This suggests that in our overweight/obese diabetic cohort, metabolic factors may overshadow gender-based differences. MASLD exhibits distinct gender-specific patterns in prevalence, severity, and associated risk factors, as highlighted by recent research. Nagral et al. (2022) highlight that men tend to have higher NAFLD rates, potentially due to greater visceral fat accumulation, which is a known risk factor for liver fat deposition [18]. Building on this, Hashimoto and Tokushige (2011) reported 2-3 times more prevalent in men, particularly among younger age groups [19]. Qiu et al. (2023) shift the focus to liver enzyme markers, such as ALT, and their gender-specific associations with NAFLD [20] and identify distinct patterns in how these markers correlate with NAFLD risk in men and women. For instance, elevated ALT levels may serve as a more reliable indicator of NAFLD in men than in women, reflecting gender differences in liver enzyme activity and NAFLD pathophysiology. Collectively, these studies reveal a consistent pattern that men generally face a higher NAFLD burden in younger age groups, driven by factors like visceral fat and metabolic differences, while women’s risk increases post-menopause, potentially due to hormonal changes. The interplay of age, hormones, and lifestyle underscores the complexity of NAFLD’s gender distribution. The age distribution differed significantly between MASLD and non-MASLD groups. Similar age-related patterns have been reported in prior studies, where MASLD risk peaks in middle age due to cumulative metabolic exposure.

Obesity and metabolic risk factors

Obesity emerged as a significant modifiable risk factor in our analysis. The mean BMI in the MASLD group was significantly higher (29.10 kg/m²) than in the non-MASLD group (26.96 kg/m²). Additionally, 33.3% of the MASLD group were classified as obese (BMI ≥30), compared to only 7.1% in the non-MASLD group (p < 0.01). This confirms existing evidence from van der Poorten D et al. (2008) and Pickhardt et al. (2012), who demonstrated that increased adiposity, particularly visceral fat, correlates strongly with hepatic fat accumulation [21,22].

Interestingly, studies such as Eslam et al. (2020) have highlighted the existence of "lean NAFLD" in Asian populations, with up to 25% of NAFLD patients showing normal BMI but high central obesity and metabolic dysfunction [23]. Our study did not extensively evaluate the waist-hip ratio or visceral fat, limiting deeper insight into this phenomenon. Future studies should incorporate these anthropometric markers. Dyslipidemia also showed a significant association with NAFLD in our cohort. Elevated total cholesterol (≥200 mg/dL) was present in 47.1% of NAFLD patients vs. 14.1% of non-NAFLD patients (p < 0.001). Furthermore, although low HDL was prevalent in both groups, it was slightly higher in the non-NAFLD group (75.8%) compared to the NAFLD group (50.9%). This paradox may reflect the combined effects of statin therapy, dietary differences, or methodological variation in defining HDL cutoffs. The role of dyslipidemia, independent of BMI, in NAFLD pathogenesis is increasingly recognized. Elevated LDL and triglycerides contribute to hepatic fat synthesis via the sterol regulatory element-binding protein-1c (SREBP-1c) pathway, promoting steatosis even in the absence of overt obesity.

Liver enzyme profiles

MASLD patients demonstrated significantly elevated serum transaminases in our study. Mean SGPT (ALT) and SGOT (AST) values were 37.77 IU/L and 43.42 IU/L, respectively, with 17.6% and 51% of patients crossing the diagnostic threshold of ≥45 IU/L. These findings are in concordance with Xuan et al. (2006), who proposed that mild ALT elevation is often the earliest biochemical sign of NAFLD. Elevated AST/ALT ratios, while more indicative of advanced fibrosis, were not explored in our dataset and warrant future analysis [24].

However, we found no significant elevation of ALP, contrasting with other studies. Like, a study by Ali et al. (2021) involving 210 obese patients found that ALP is an independent predictor of significant fibrosis (OR 1.03, 95% CI 1.01-1.05), with an AUROC of 0.845 [25]. Similarly, a study by Bazick et al. (2015) of 346 diabetic NAFLD patients developed a clinical model including ALP to predict advanced fibrosis (OR 1.014, 95% CI 1.005-1.024), achieving an AUROC of 0.80 [26]. Additionally, a study by Pantsari et al. (2006) of 135 NAFLD patients indicated that older females with isolated ALP elevation may have steatohepatitis, with nearly one-third showing advanced fibrosis [27].

Strengths of the study

This study is among the few region-specific investigations into the prevalence and determinants of MASLD in urban diabetic populations in Eastern India. It utilizes a well-defined cohort with standardized data collection methods and includes comprehensive biochemical profiling to assess metabolic risk factors. The findings offer valuable insights into the interplay between glycemic control, obesity, dyslipidemia, and liver health in an underserved demographic.

Limitations of the study

Our study has several limitations. First, only patients with BMI ≥25 kg/m² were included; therefore, results apply only to overweight/obese diabetics. Second, ultrasound-while practical-may miss mild steatosis and cannot stage fibrosis. Third, important covariates such as waist circumference, diabetes duration, and medication history, including statin use, were not comprehensively collected. Fourth, purposive sampling may introduce selection bias and reduce generalizability. These limitations should be considered when interpreting the findings.

Future directions

Future research should focus on large-scale, multicenter longitudinal studies to better understand the natural history and progression of MASLD in Indian diabetics. Incorporating non-invasive fibrosis markers, genetic testing (e.g., PNPLA3), and advanced imaging techniques can enhance diagnostic accuracy. Public health research is also needed to evaluate the effectiveness of community-based lifestyle interventions tailored to high-risk populations.

Key recommendations

Key recommendations for addressing MASLD in India include universal screening of high-risk groups such as all T2DM patients with BMI ≥25 kg/m² and non-obese diabetics with central adiposity, using tools like abdominal ultrasound, liver enzymes, lipid profiles, and non-invasive fibrosis scores. Early intervention is crucial, targeting HbA1c <7%, promoting 5-10% weight loss, ≥150 minutes of aerobic activity per week, and a high-fibre, low-GI diet. Pharmacological options like pioglitazone and GLP-1 receptor agonists may be used judiciously. Public health efforts should focus on awareness campaigns, community programs, and policy changes under NPCDCS to integrate liver screening into diabetic care. Finally, India-specific research should explore disease progression, validate diagnostic tools, and study genetic markers for better risk prediction.

A call to action

MASLD is not merely a hepatic disorder but a systemic, silent threat embedded in India’s metabolic landscape. Proactive integration of MASLD screening into routine diabetes care, combined with population-level lifestyle interventions and continued research, is essential to reverse this growing tide. Failure to act may result in escalating rates of cirrhosis, liver failure, and cardiovascular mortality-burdens that the Indian healthcare system is ill-equipped to manage without timely reform.

## Conclusions

MASLD affected more than one-third of overweight and obese T2DM patients in this Eastern Indian cohort. Its close association with poor glycemic control, obesity, and dyslipidemia underscores its importance as a metabolic complication in diabetes care.

Given the largely asymptomatic nature of MASLD and its potential long-term consequences, routine screening in high-risk diabetic patients should be prioritized. Integrating early detection with lifestyle and pharmacological interventions can reduce disease progression and healthcare burden.
